# Spanish multicenter real – life registry of retrievable vena cava filters (REFiVeC)

**DOI:** 10.1186/s42155-020-00114-5

**Published:** 2020-05-18

**Authors:** Miguel A. De Gregorio, Jose A. Guirola, Jose Urbano, Ignacio Díaz-Lorenzo, Jose J. Muñoz, Elena Villacastin, Antonio Lopez-Medina, Ana L. Figueredo, Javier Guerrero, Sergio Sierre, Javier Blazquez Sanchez, William T. Kuo, David Jimenez

**Affiliations:** 1 Hospital Universitario Lozano Blesa, Universidad de Zaragoza, Zaragoza, Spain; 2 Hospitales Vithas, Madrid, Spain; 3 Hospital Universitario La Princesa, Madrid, Spain; 4 Hospital Universitario Carlos Haya, Malaga, Spain; 5 Hospital Rio Hortega, Valladolid, Spain; 6 Hospital de Basurto Hospital, Bilbao, Spain; 7 Hospital Universitario Miguel Servet, Zaragoza, Spain; 8 Grupo Investigación Técnicas Mínimamente Invasivas (GITMI), Zaragoza, Spain; 9 Hospital Universitario Austral, Buenos Aires, Argentina; 10 Hospital Ramón y Cajal, Madrid, Spain; 11 Department of Radiology, Stanford University Medical Center, Stanford, CA USA; 12 Hospital Ramón y Cajal and Universidad de Alcalá (IRYCIS), Madrid, Spain

**Keywords:** Inferior vena cava filters, Retrievable filters, Venous thrombotic disease (VTD)

## Abstract

**Background:**

The treatment of venous thromboembolic disease the treatment of choice is systemic anticoagulation. However, the interruption of the inferior vena cava with filters has been recommended when anticoagulation fails or there is a contraindication. Due to the rising inferior vena cava filter (IVCF) complications, physicians are encouraged to retrieve them when there is no longer recommended. In daily practice, it may be a difficult close follow-up of these patients. In this study, the primary objective was to evaluate the IVCF retrieval rate of all implanted filters in a Spanish registry. Secondary objectives were to analyze the causes of failed retrieval, procedure-related complications, and outcomes at a 12-month follow-up.

**Results:**

Three hundred fifty-six vena cava filters were implanted in 355 patients. The types of filter were: Gunther Tulip (Cook Medical) 160 (44.9%), Optease (Cordis) 77 (21.6%), Celect (Cook Medical) 49 (13, 7%), Aegisy (Lifetech Scientific) 33 (9.2%), Option ELITE (Argon Medical devices) 16 (4.4%), Denali filter (BD Bard) 11 (3.08%), ALN filter (ALN) 10 (2.8%).

Removal was achieved in 274/356 (76,9%). eighty-two (23,1%) IVCF were not retrieved due to the following: 41 (11,5%) patients required ongoing filtration, 24 IVCF (6,7%) patients died before retrieval, and 17 (4,7%) impossibility of retrieval because of a tilted and embedded filter apex. There were no major complications observed.

**Conclusions:**

The global retrieval rate of IVCF was achieved in 76.9%, and the adjusted retrieval rate was of 94.15% with no major complications. IVCF tilting was associated with failure of filter removal in less than 5% of cases. This study demonstrates that the retrieval procedure of IVCF is controlled by the clinician and not by the interventional radiologist.

## Background

Venous thromboembolic disease (VTD) is a serious disease that affects 1–2 per 1000 of European citizens (Monreal et al. 2015). VTE includes acute deep vein thrombosis (DVT) and pulmonary embolism (PE). The treatment of choice in VTD is therapeutic systemic anticoagulation which includes the treatment with: heparin, low weight heparins, warfarin, acenocoumarol or the new oral anticoagulants (apixaban, endoxaban, rivaroxaban, or dabigatran) (Pattullo et al. 2016). When anticoagulation fails or is contraindicated, inferior vena cava interruption is recommended, historically it was performed via surgery, and over the past few decades, the endovascular procedure is the standard of choice using various implantable filtration devices with almost in all devices with a retrievable option (Yunus et al. 2008).

Until the publication of H Decousus, et al. in 1998 (Decousus et al. 1998), even with little clinical evidence, no one questioned the usefulness of inferior vena cava filter (IVCF) for the prophylaxis and treatment of VTD (Proctor and Greenfield 2008; Athanasoulis et al. 2000; Stein et al. 2004; Dalen and Stein 2013). It was Decousus, et al. and the PREPIC study (PREPIC Study Group 2005) that demonstrated that IVCF represents a potential benefit with protection against life-threatening pulmonary embolism in a short-term; however, with a higher risk of symptomatic DVT in a long-term without mortality difference at 8 years of follow-up. Therefore, the use of IVCF decreased in Europe (PREPIC Study Group 2005; Reddy et al. 2017; Wadhwa et al. 2017). The arrival of new retrievable IVCF increased the indications for filter placement, to provide protection against PE in the short-term while avoiding the long-term DVT risks by removing the filter in a short period. In the past decade, it has encountered a rising complication in the IVCF placement reported in the United States, MAUDE database (Amendola and Acosta 2016), that resulted in a Safety Alert issued by the FDA in 2010 (Morales et al. 2013). This safety alert encourages physicians and clinicians responsible for the care of patients with IVCF, to consider removing the filters when there was no longer an indication of inferior vena cava interruption. (Amendola and Acosta 2016; Food and Drug Administration 2011) However, in daily clinical practice, it may be difficult to follow patients closely and prompt filter removal.

We present the results of a multicenter prospective registry involving 15 tertiary Spanish hospitals using a protocol to facilitate close patient follow-up for prompt filter removal.

The primary objective of this study was to evaluate all the inferior vena cava filter (IVCF) retrieval rate of implanted IVC filters. Secondary objectives were to analyze the causes of failed retrieval, procedure-related complications, and outcomes at a 12-month follow-up.

## Material and methods

The SERVEI-REFiVeC (Registro Español de Filtros en Vena Cava Inferior) Registry is a prospective multicenter study endorsed by the Spanish Society of Vascular and Interventional Radiology (SERVEI) and by the Zaragoza University. This Registry received institutional review board approval in May 2016 (CP-CI number PI16 / 0142), and the study was registered in Clinical Trial Gov (NCT02757001). The registry was open from 01 to 04-16 until 01–04-18. The form was designed by the research group (GITMI) of the University of Zaragoza.

An electronic patient report form was specifically designed for this registry and hosted on the Spanish Society of Vascular and Interventional Radiology (SERVEI), webpage (Sociedad Española Radiología Intervencionista (SERVEI), estudios y registros 2020).

This was open to all members of SERVEI society, after identification and acceptance by the study principal investigator (PI), and all of the interventional radiologists (IRs) had at least 5 years in experience for the placement and retrieval of IVCF. Over 2 years, all patients with an IVCF implantation were consecutively enrolled by an electronic registry. The following data were gathered: demographics, VTE risk factors, filter placement indications, IVCF type, filter dwell time, number of retrieval attempts and retrieval outcome (Table 1). The follow-up was carried out at least 1 year and determined if an appearance of PE or DVT. In case of clinical suspicion of PE or DVT, pulmonary CT angiography was performed for the suspicion of PE or ultrasound- Doppler for DVT. Complications were classified according to CIRSE standards (Filippiadis et al. 2017). The study was conducted following the “Strengthening the Reporting of Observational Studies in Epidemiology (STROBE) Statement: guidelines for reporting observational studies (von Elm et al. 2007).

**Table 1:** Registry form. Data points included in the registry

Identification	Number
	Sex
	Age
	Risk factors
	Filter indication
Technical data	Access
	Filter type
	Filter implantation date
Recovery attemps	1,2,3 …
Death before recovery	Yes /NO
Anticoagulant treatment	Yes /NO
Recovery access	Yugular vein (R/L)
	Femoral vein (R/L)
	Others
Recovery with manufacturer recommended set	Set recommended by the manufacturer
Recovery with other maneuvers	Snare, ballooms, cocodrile clips, grasping
Cause of inability to filter recover	Tilt, leg penetration, fibrosis, thrombosis, death, others
Follow-up	3, 6, 12 months
	Recurrence pulmonary embolism, other causes

### Statistical analysis

We used chi-square or Fisher’s exact tests to compare categorical data between groups. We used the Shapiro-Wilk test to assess continuous data for a normal distribution.

We used two-tailed unpaired t-tests to compare parametric continuous data between two unpaired groups, and we used the Mann-Whitney U test for non-parametric data comparisons. We conducted statistical analyses using STATA version 13.1 (STATA Corp, College Station, Texas). All hypothesis tests were two-sided, with a significance level of 0.05.

The registry was designed to enroll at least 200 patients with IVCF and recorded clinical follow-up at least one year after recovery, evaluating possible episodes of recurrent PE, DVT or possible death of the patient.

## Results

### Demographics

From April 2016, until April 2018, three hundred fifty-six vena cava filters were implanted in 355 patients (1 patient received two filters for having double vena cava). The mean age was 59.7 ± 13.7 years (range 89–19 years. One hundred and eighty-two (51.1%) patients were men and 174 (48.8%) women, 15 Spanish tertiary hospitals participated in the REFiVeC registry. The average IVCF implantation in each center was 23.6 ± 25.9 (range 101–1 IVCF). The types of filter used in the registry were: Gunther Tulip (Cook Medical) 160 (44.9%), Optease (Cordis) 77 (21.6%), Celect (Cook Medical) 49 (13, 7%), Aegisy (Lifetech Scientific) 33 (9.2%), Option ELITE (Argon Medical devices) 16 (4.4%), Denali (BD Bard) 11 (3.08%), ALN filter (ALN) 10 (2.8%).

### Indications

VTD risk factors included immobilization in 197 (55.4%), neoplasm in 55 (15.4%), recent surgery in 46 (12.9%), history of VTE in 35 (9.8%) and contraceptive use in 24 (6.7%). Of the patients who required a vena cava filter, 309 had one risk factor for VTD, 43 had two risk factors, and 4 had more than two risk factors. The main indications for IVCF were: presence of VTE with contraindication to anticoagulation in 188 (52.8%), Prevention in high-risk patients with DVT in 120 (33.7%), (in this case, 54 patients suffered massive PE, 49 suffered iliofemoral DVT and 17 COPD and DVT), and recurrent PE despite anticoagulation in 48 (13.4%) (Table 2).

**Table 2:** The main indication for Inferior Vena Cava Filters

Indications	Direct cause	n	N	%
Prevention in high risk patients with DVT	- EP massive treated with fibrinolysis o thrombectomy	54	120	33,7
	- Iliofemoral DVT	49		
	- COPD + DVT	17		
Contraindication for anticoagulation	- Recente bleeding	104	188	52,8
	- Recent surgery	42		
	- Brain tumor /Recent stroke	27		
	- Severe trauma	15		
Recurrence of pulmonary embolism while receiving anticoagulation			48	13,4

### Access and imaging

The access route used for the implantation of the filter was: the right internal jugular vein in 180 cases (50.5%), the right femoral vein in 148 (41.5%), the left femoral vein in 25 (7.02%) and the left internal jugular vein in 2 (0.5%) cases. Cavography was performed in all patients during filter removal, 260 (73.03%) patients underwent abdominal computed tomography before IVCF retrieval. The indication of abdominal CT was to facilitate withdrawal (knowing the inclination, the presence of thrombosis or migration) and was performed on the same day before the IVCF retrieval. The abdominal CT, 185 (71.1%) patients had normal findings regarding the position and tilt of the filter; in 41 (15.7%) patients tilting of the IVCF was appreciated < 15° with the IVC, in 12 (4.6%) patients IVCF tilt was found > 15°. Penetration of the legs in the IVC > 3 mm was appreciated in 2 (0.76%) patients, inclusion of the superior hook in the wall of the IVC or some other element of the filter was observed in 16 (56.1%) patients, and in 4 (1, 5%) patients had evidence of thrombosis of the IVCF. Three hundred and one (84.5%) patients were receiving anticoagulation therapy at the time of filter removal, and 55 (154%) patients were not anticoagulated.

### Retrieved IVCF

**Fig. 1:**
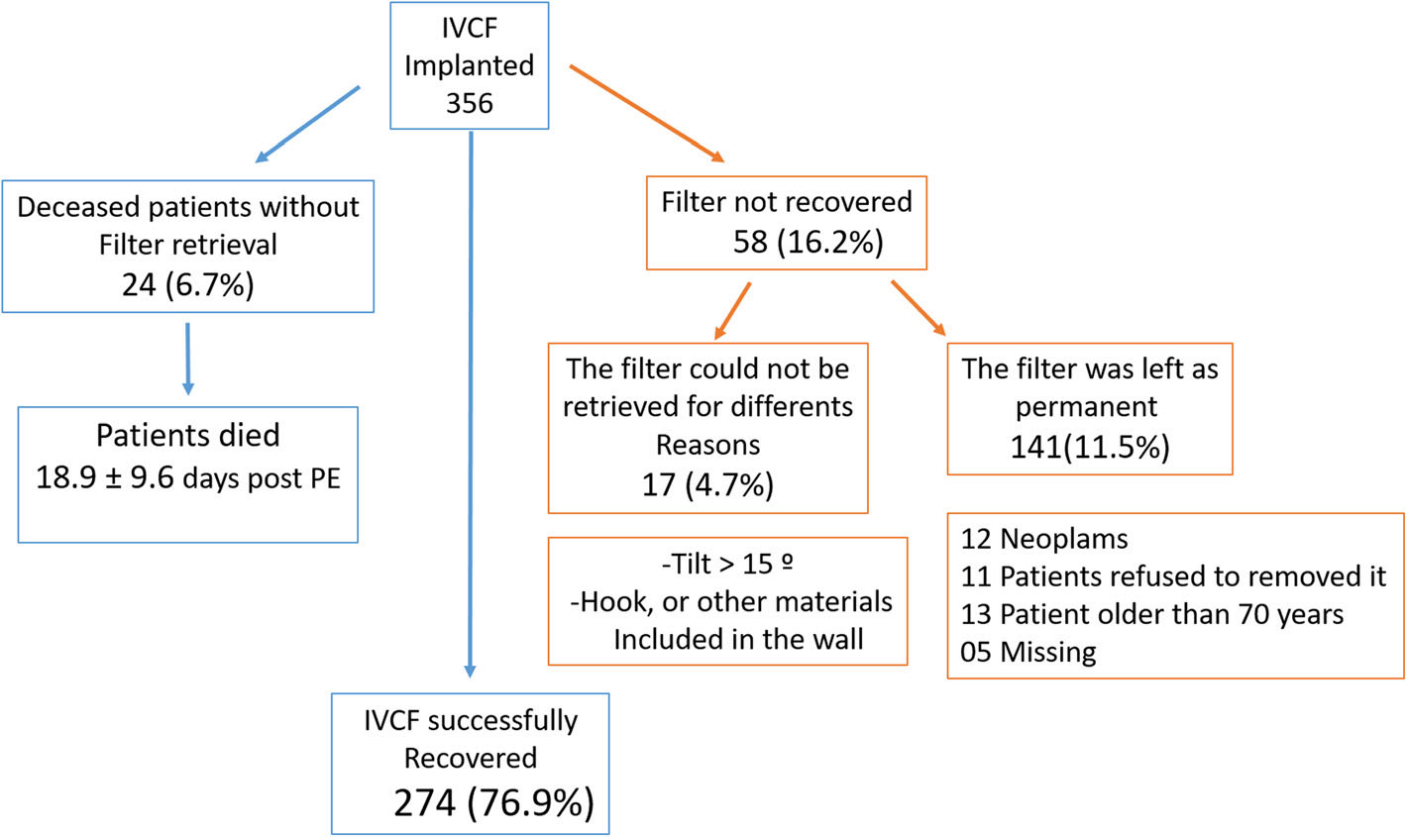
Diagram showing the distribution of inferior vena cava filters. Filters retrieved successfully and unable to retrieve filters with their causes

A total of 274/356 (76.9%) filters were successfully removed. Figure 1 shows the distribution of the retrieved IVCF. In eighty-two (23,0%) patients, the filters were not removed, in 41 patients (11,5%) the retrievable filters were left in as permanent filters (12 patients with advanced neoplasm, 11 patients refused removal, 13 patients were older than 70 years and ongoing filtration was desired due to comorbidities, and five patients were lost to follow up). In twenty-four (6,7%) patients, the IVCF was not removed since the patients died during the follow-up interval at a mean of 18.9 ± 9.6 days (range 2–36 days). Retrieval was not possible in seventeen patients (4,7%) due to a tilted (> 15 °) and embedded filter apex. Table 3 shows the IVCF dwell time. The mean dwell time was 44.8 ± 170.4 days, a median of 31 days (ranging 1–2920 days). Table 4 shows the number and the percentage of each type of filters that were not recovered, as a result of the patients had died, could not be retrieved or because they were left as permanent filters.

**Table 3:** Days until the filter was retrieved in each type of filter, either satisfactorily or failed as well as the range (maximum and minimum days for each type of filter)

Filter (356)	N	%	Mean days until retrieval		Range days		p
			Successful	Failed	Min	Max	
Gunther Tulip (Cook)	160	44,9	146/64,6	2/87,5	1	2920	< 0.01
Optease (Cordis)	77	21,6	44/29,1	11/48,09	12	98	< 0.01
Celect (Cook)	49	13,7	42/ 41,1	2/151	19	270	< 0.01
Aegisy (Lifetech	33	9,2	20/14,9	1/13	11	27	0.05
Option ELITE (Argon)	16	4,4	11/32,3	1/28	26	41	0.01
Denali (Bard)	11	3,08	8/31	0/0	26	35	0.04
ALN Filter (ALN)	10	2,8	8/32,3	0/0	26	366	0.04
TOTAL	356				1	2920

**Table 4:** Distribution by type of filter (Successful withdrawal or failure, as well as filters that could not be removed because the patient died during their hospital stay or for various reasons, were left as permanent filters)

Filter	N	%	Successful	Failed	Dead*	Permanent	p
Gunther Tulip (Cook)	160	44,9	146 /91,2%	2/1,2%	9/5,6%	2/1,2	< 0.01
Optease (Cordis)	77	21,6	42/54,5%	11/14,2%	6/7,7%	17/22,0%	< 0.01
Celect (Cook)	49	13,7	42/85,7%	2/4,0%	2/4,0%	2/4,0%	< 0.01
Aegisy (Lifetech	33	9,2	20/60,6%	1/3,0%	3/9,0%	12/36,3%	< 0.1
Option ELITE (Argon)	16	4,4	11/68,7%	1/6,25%	1/6,2%	4/25%	0.05
Denali (Bard)	11	3,08	7/63,6%	0/0	1/9,0%	3/27,7%	0.04
ALN Filter (ALN)	10	2,8	6/60%	0/0	2/20	1/10%	0.04
	356		274	17	24	41	

* Patients died before inferior vena cava filter was scheduled for retrieval

### Difficulties and complications

Removal difficulties occur in 26 patients (7.3%) (mainly related to tilting > 15 ° and inclusion of the upper hook or some structure in the wall of the IVC.

**Fig. 2:**
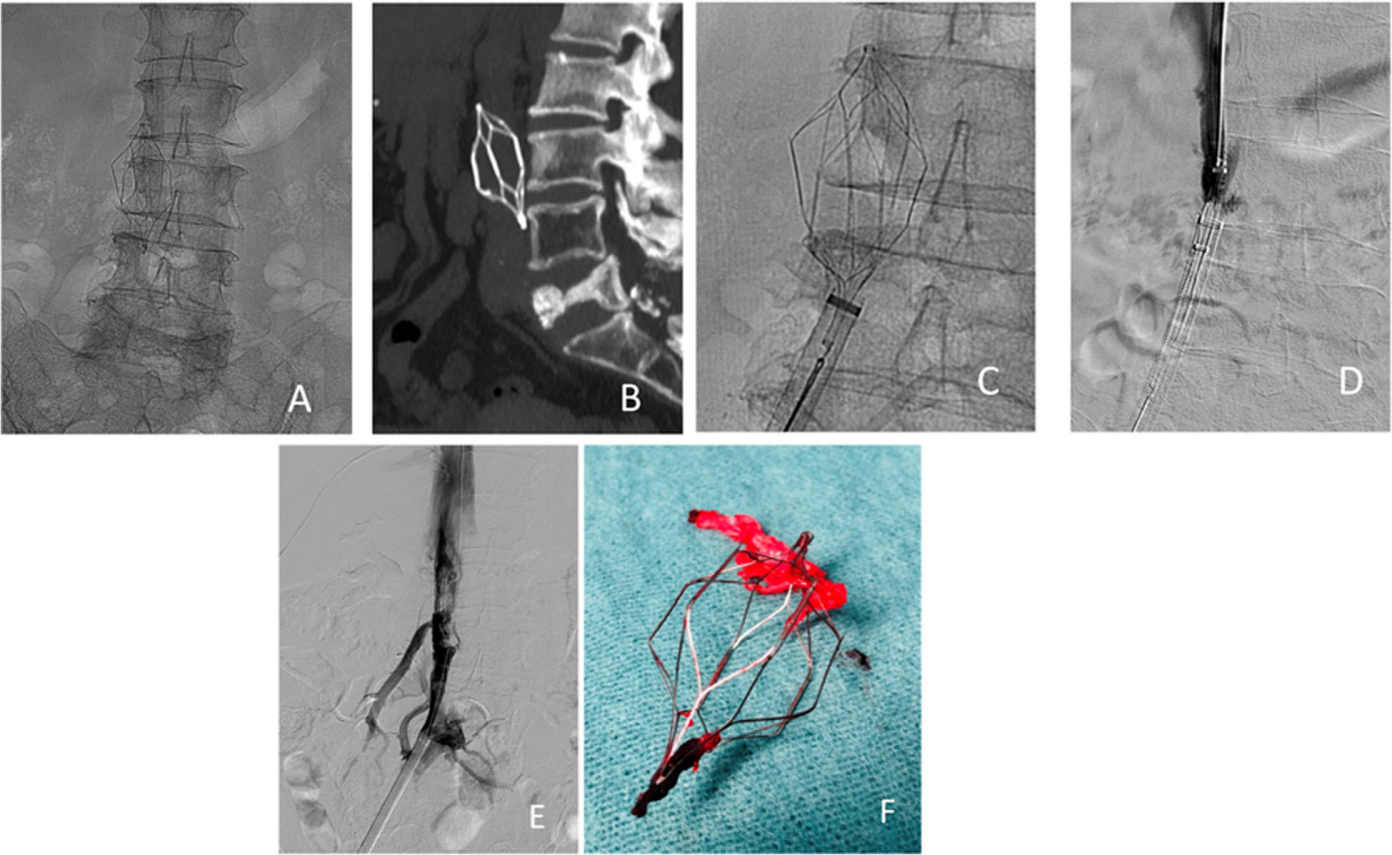
Example of IVCF retrieval with difficulty. A 61-year-old female patient diagnosed with multiple myeloma. Six years ago she had DVT in her left lower limb. She was treated with unfractionated heparin and had an upper-GI bleed, and anticoagulants became contraindicated. An Optease filter was implanted infrarenal. **a**. abdominal x-ray showed the Optease filter in a proper position. **b**. Abdominal CT with sagittal MPR showed the filter slightly tilted on the vertical axis. **c**. Femoral access with a 16 Fr sheath and a 25 mm snare (Amplatz GooseNet snare). After several attempts, it was impossible to recover it. **d**. Jugular access was made with a 12 F sheath and a guide was passed through the upper vertex of the filter and with several movements from both accesses, the IVCF was introduced into the sheath and the filter could be recovered. **e**. Cavography after filter retrieval (shows endothelial alteration). **f**. The filter recovered with endothelial remains

Special endovascular maneuvers were performed to retrieve filters with an average of 2.8 maneuvers, with a range of 1–4 additional maneuvers (femoral-jugular double access, Snare-Loop Technique, the Hangman Technique) and repeated attempts at recovery in 9/26 patients, IVCF was recovered with an average of 2.1 attempts (range 1–3). Despite several attempts (average 2.5 and 1–3 range) in 17/26 patients, IVCF could not be recovered and was left as permanent filters (Fig. 2) (Table 5). Of the IVCF defined as retrievable filters (without patients who died and those who for various reasons were left as permanent filters), the global removal success was 76.96%. When comparing the dwell time of the filters extracted successfully and in which it failed, the average period was 35.2 ± 15 days with a median 32 days for the extracted ones versus 65 ± 56 days on average with a median of 48 days in the that failed (*p* = 0.0011). IVCF thrombosis was observed in four patients (1.2%). After fibrinolysis in two patients and thromboaspiration in 2, the filter could be removed without any complications. Complications were found in 12 patients (3.3%): 6 neck hematomas and 5 groin hematoma, (type I complication) (Filippiadis et al. 2017). Accidental carotid punctured in 1 patient, which required compression and admission of 24 h (type II complication) (Filippiadis et al. 2017). In 276 (77.5%) patients, fluoroscopy time and Air Kerma were studied (Table 6).

**Table 5:** Main causes of IVCF recovery failure and number retrieval attempts

Filter	N	Failed	Reasons for failed	Recovery Attemps
Gunther Tulip (Cook)	160	2/1,2%	-Tilt> 15°, Hook included in IVC wall	2
			- Hook and legs included in IVC wall	3
Optease (Cordis)	77	11/14,2%	- Legs and structures included in IVC wall	2 (5 pats)
			- Legs and structures included in IVC wall	1 (6 pats.)
Celect (Cook)	49	2/4,0%	-Tilt> 15°, Hook included in IVC wall	2
			- Tilt> 15°, Hook included in IVC wall	1
Aegisy (Lifetech)	33	1/3,0%	- Legs and structures included in IVC wall	1
Option ELITE (Argon)	16	1/6,25%	-Tilt> 15°, Hook included in IVC wall	2
Denali (Bard)	11	0/0	–	
ALN Filter (ALN)	10	0/0	–	
	356	17		

(Pats: Patients)

**Table 6:** Fluoroscopy time and Air Kerma data for retrieval procedure (successful and failed filter retrieval)

Recovery		Fluoroscopy time Minutes	Air Kerma mGy	p
Successful	Mean	13,7	395,2	< 0.01
	SD	11,6	505,7	
	Max	33,1	1796,1	
	Min	2,5	94,7	
Failed	Mean	41,9	1641,7	< 0.01
	SD	11	421	
	Max	64	2117,2	
	Min	25,4	1314,9	

### Follow-up

In 264 (74.1%) patients, follow-up time was 11.3 ± 3.2 months after IVCF recovery. All patients were treated with oral anticoagulation or low molecular weight between 6 and 8 months. There were 2 cases of recurrence of PE after IVCF retrieval. Both were documented with pulmonary CT angiography, echocardiography, and biological markers. One of them, occurred when the patient was not anticoagulated, and was classified as a low-intermediate risk for PE and was treated with new oral anticoagulant treatment. The other, a young patient, diagnosed with hematologic disease and hereditary thrombophilia (homologous Leiden Factor V) was decided on the new placement of definitive filter and anticoagulation treatment for life.

## Discussion

The SERVEI-REFiVeC registry is a study of 356 retrievable ICVFs in 355 patients, which gathers the activity of fifteen major Spanish hospitals. This study has limitations since in Spain, there is no consensus, protocol or guidelines accepted by the different societies regarding VTD regarding placement and retrieval of IVCF. Interventional Radiologists are aware of the recommendations of the FDA and other administration to remove all filters as soon as possible, once they are no longer needed. The total recovery rate of FVCI in this study was 76.9%, it might seem low if we compare it with the data from the CIRSE Registry (De Gregorio et al. 2006) in which the recovery rate is 92%. Other non-multicenter studies (De Gregorio MA et al.) (Sarosiek et al. 2013) reach 96% success in recovery. However, the British Society of Interventional Radiology (BSIR) registry (Lee et al. 2015) presents similar or even lower data (426/514) 66,9%, other authors such as Sarosiek et al. in 2013 (de Gregorio et al. 2018) in a series of 679 retrievable filters only 58 (8%) were removed.

The differences can be very important because the study designs are different. In the present SERVEI-REFiVeC study, after withdrawing patients which had died 24(6%), and 41 (11.5%) patients with the initial intention to retrieve the filter, but finally for different reasons or because there is no clear protocol in their hospital, the petitionary clinician decided not to remove the filter. The adjusted retrieval rate of Vena Cava Filters in SERVEI-REFiVeC reaches 94.15% (291/356). Only in 17 patients (4.7%) was attempted for retrieval but was not possible for different reasons.

Several studies have shown that the retrievability of different types and models of filters is similar and there are no significant differences (Sarosiek et al. 2013; Uberoi et al. 2013a; Lyon et al. 2009; Rimon et al. 2009; Pellerin et al. 2008; Uberoi et al. 2013b; Deso et al. 2016; Kuo et al. 2012). The importance of the retrievable filters is recoverability property, because it is a thrombogenic device, that according to various studies provides it limited clinical benefit and can cause complications (Decousus et al. 1998; PREPIC Study Group 2005; Amendola and Acosta 2016).

It seems that the main difficulty for the recovery of these retrievable filters is the tilting and the embedded apex which may result in fibrosis or endothelialization of the device components into the wall (Uberoi et al. 2013a; Lyon et al. 2009). These factors could be prevented by the operator by improving the implantation technique, and the dwell time of the device. It is important an advanced technique in the implantation of the filter, avoiding tilting > 15°. On the other hand, the FDA recommends the removal of the filters as soon as possible when vena cava interruption is no longer needed. This logical recommendation, together with the operator’s responsibility, is to suggest the referring physician and the patient retrieve the filter as soon as possible and avoid retrievable procedures with long dwelling time which may present more difficulty and possible complications.

The advanced skills of the operator must not be only evident in the implantation of the filter, but also be able to manage different devices and additional retrieval maneuvers (Iliescu and Haskal 2012; Desai et al. 2017). In this study, additional maneuvers were used in 33 (9.2%) patients. Despite these additional maneuvers, half of the cases (17 patients / 4.7%) the filters could not be removed.

Prolonged filter dwell time has been associated with a more difficult recovery of the filter, and an increase in complications secondary to the retrieval procedure. The complications include: device fracture, migration, organ penetration by device components, and an elevated risk of thrombosis / DVT (Andreoli et al. 2014). MAUDE (Manufacturer and User Facility Device Experience) (White et al. 2013) has published a high rate of complications in the handling of retrievable filters, which has led the FDA in this same year to do a serious communication insisting to withdraw the FVCI as soon as possible. However, the main filter recovery records do not reveal significant and serious complication rates (Sarosiek et al. 2013; Uberoi et al. 2013a; Deso et al. 2016). In this study, complications related to the access route in the jugular vein were found, the most important an accidental puncture of the carotid artery. There was also no relationship between the difficulties of IVCF recovery and the filter dwell time.

Recent studies have questioned the efficacy of IVCF (Dalen and Stein 2013). Some studies have been pointed out the controversial role of IVCF in the treatment of the VTD. The existence of great variability in the use of filters between hospitals with similar indications of 0% to 38% is known (Prasad et al. 2013). Some authors doubt the effectiveness of IVCF in the protection against PE since in a series of 504 patients with PE and IVCF 7.8% presented recurrence of PE despite the filter (de Gregorio et al. 2018; Kearon et al. 2012). The guidelines of the American College of Chest Physicians (ACCP) and the European Society of Cardiology (ESC) in their recommendations reserve the use of IVCF for the failure and/or contraindication of anticoagulation (Konstantinides et al. 2019; Kearon et al. 2016).

The PREPIC 1 study (Decousus et al. 1998) reinforced by the PREPIC 2 study, (PREPIC Study Group 2005) based on IVCF, initially it decreases PE but increases the rate of DVT and does not influence the mortality of VTD. Current guidelines do not recommend IVCF in anticoagulated patients with VTD (Recommendation 1B) (Dalen 2012).

Even though retrievable filters have largely replaced permanent filters, there is still not enough clinical evidence to increase IVCF indications and modify current guidelines (Uberoi et al. 2013a). The use of a retrievable IVCF with a prompt removal is theoretically consistent with the conclusions of both PREPIC studies (Decousus et al. 1998; PREPIC Study Group 2005). Recoverable filters could potentially be effective in the prevention of life-threatening PE in the short-term. IVCF should be removed as soon as possible when their presence is no longer needed, and avoid filter-related complications including long-term post-thrombotic syndrome. In this study, the 2016 ACCP filter guidelines (Kearon et al. 2016) were closely followed and the indications for filter placement were absolute in 66.2%, in contrast to the CIRSE Registry where absolute indications were only 40% (Uberoi et al. 2013a).

Retrievable IVCF has demonstrated efficacy in patients with unstable PE (Dalen and Stein 2016; Stein et al. 2012). In patients with unstable PE, hospital mortality was 18% in patients with fibrinolytic therapy alone, while hospital mortality was reduced to 7.6% when IVCF was used (*p* < 0001) (Stein et al. 2012). However, despite the big difference in the number of filters implanted per capita in the US versus Europe, the overall death rate from PE in both populations remains similar (Uberoi et al. 2013a).

There are important limitations of this study, even though the study was supported by SERVEI, only 15 hospitals participated, and Spain has a total of 118 hospitals with IR (De Gregorio and Urbano 2017). The number of participants was small and does not resemble the absolute truth of the implantation of IVCF in Spain. In our opinion, there are too many IVCF left as permanent filters (41/11.5%). The involvement of the interventional radiologists in the decision to remove or leave the IVCF as permanent is unknown. However, the study objective was not to show the retrieved filters but implanted filters and their possibility of recovery, which shows us a real situation within a small sample. A guide or clinical consensus for the management of IVCF should be promoted from the scientific societies regarding VTD. Finally, it would be necessary to perform a multicenter, and multinational registry in Europe to determine the exact use of IVCF and their retrieval rate.

## Conclusion

In conclusion, IVCF retrieval was achieved > 75% in a cohort with no major procedural complications. IVCF tilting and embedded apex was associated with failure of filter removal in less than 5% of cases. This paper does not demonstrate a good correlation between the difficulty of the IVCF recovery and the dwell time. The global retrieval rate was of 76.9%, however the adjusted retrieval rate was of 94.15% comparable with recent studies. This study demonstrates that the retrieval procedure of IVCF is controlled by the clinician and not by the interventional radiologist.

## Data Availability

The data used and/or analyzed during the current study are available from the corresponding author on reasonable request.

## Abbreviations

ACCP: American College of Chest Physicians
BSIR: British Society of Interventional Radiology
DVT: Deep vein thrombosis
ESC: European Society of Cardiology
IVCF: Inferior vena cava filters
MAUDE: Manufacturer and User Facility Device Experience
PE: Pulmonary embolism
SERVEI: Spanish Society of Vascular and Interventional Radiology
VTD: Venous thromboembolic disease
